# Molecular Mechanisms of Islet Amyloid Polypeptide Aggregation: Towards Chemical Strategies to Prevent Amyloid Formation and to Design Non-Aggregating Peptide Therapeutics

**DOI:** 10.3390/ijms27062598

**Published:** 2026-03-12

**Authors:** Cécile Bousch, Frédérique Bérubé, Margaryta Babych, Sandrine Ongeri, Steve Bourgault

**Affiliations:** 1 Department of Chemistry, Université du Québec à Montréal, C.P. 8888, Succursale Centre-Ville, Montreal, QC H3C 3P8, Canada; bousch.cecile@courrier.uqam.ca (C.B.); berube.frederique@uqam.ca (F.B.); margaryta.babych@mcgill.ca (M.B.); 2 Quebec Network for Research on Protein Function, Engineering and Applications, PROTEO, Montreal, QC H2X 3Y7, Canada; 3 BioCIS, CNRS, Université Paris Saclay, 17 Avenue des Sciences, Bâtiment Henri Moissan, 91400 Orsay, France

**Keywords:** IAPP, islet amyloid polypeptide, amylin, aggregation, amyloid fibrils, conformational conversion, oligomers, self-assembly, GPCR agonists

## Abstract

The islet amyloid polypeptide (IAPP) is a peptide hormone playing key biological roles, including glucose homeostasis and regulation of food intake, conferring high therapeutic potential to treat metabolic disorders. Nonetheless, IAPP is mainly known as the major component of the amyloid fibrils observed in the pancreatic islets of patients afflicted with type 2 diabetes, and the accumulation of these insoluble protein deposits correlates closely with the loss of pancreatic β-cells. The inherent aggregation propensity of this peptide hormone is not only associated with the pathogenesis of type 2 diabetes but also complicates the design of IAPP derivatives for the treatment of metabolic disorders. Accordingly, elucidating the molecular mechanisms by which IAPP self-assembles into amyloid fibrils is critical to identify chemical strategies to arrest aggregation, as well as to design safe and stable IAPP-derived therapeutics. This review aims at presenting the different mechanistic models of IAPP aggregation and how to exploit this information to identify inhibitors of amyloid formation and non-aggregating peptide agonists. After discussing the conformational conversions allowing IAPP to undergo a mainly disordered monomeric conformation into ordered cross-β-sheet quaternary supramolecular structures, we present chemical strategies to prevent amyloid deposition and to develop non-aggregating peptide-based therapeutics.

## 1. Introduction

The islet amyloid polypeptide (IAPP), or amylin, is a 37-residue peptide hormone that is co-secreted with insulin by β-pancreatic cells [1]. By activating specific G-protein coupled receptors (GPCRs), IAPP plays critical physiological roles, including regulation of glucose homeostasis [2,3], food intake [4], osteoblast proliferation, and osteoclastic bone resorption [5]. Notably, IAPP derivatives are currently used in the clinics alongside insulin to help people afflicted with type 2 diabetes (T2D) to regulate their glucose levels [6]. Nonetheless, this peptide was originally identified as the main proteinaceous component of amyloid deposits observed in the pancreatic islets of patients suffering from T2D [7,8,9]. These hyalin structures accumulating in the pancreas were already reported in 1901 [10], and their accumulation correlates closely with the progression of the disease and the loss of pancreatic β-cells [11]. The inherent aggregation propensity of IAPP is not only associated with the pathogenesis of T2D but also complicates the design and formulation of peptide derivatives for the treatment of metabolic disorders, such as diabetes and obesity [12,13,14]. Accordingly, the understanding of the molecular mechanisms by which IAPP self-assembles into an insoluble quaternary structure is critical to identify chemical tools to prevent amyloid depositions and associated cytotoxicity, as well as to design non-aggregating IAPP-derived therapeutics.

In this context, this review aims at offering an updated perspective on the mechanisms of IAPP aggregation and how this knowledge can be exploited for the identification of inhibitors of amyloid formation and for the development of non-aggregating peptide agonists. After briefly presenting IAPP, we will discuss the conformational conversion that allows the peptide to undergo a mainly disordered monomeric conformation into ordered cross-β-sheet quaternary supramolecular structures. This information will allow us to present rational strategies to inhibit amyloid formation and to develop non-aggregating peptide-based therapeutics. While several excellent and highly comprehensive reviews on IAPP amyloidogenesis and its implications in the etiology of T2D have been recently published [15,16,17], the present review aims at specifically bridging the gap between the mechanisms of IAPP amyloid self-assembly and the design of chemical strategies to arrest aggregation and design therapeutics.

## 2. Islet Amyloid Polypeptide (IAPP)

**Figure 1:**
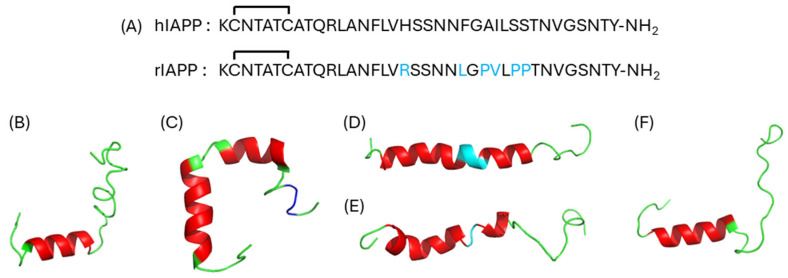
Sequences and secondary structures of IAPP. (**A**) IAPP primary sequences found in humans (hIAPP) and rats (rIAPP). Residues in rIAPP indicated in blue differ from the human sequence. (**B**–**D**) Secondary structures of hIAPP: (**B**) short helix (PDB: 5MGQ) [42], (**C**) long-and-kinked helix (PDB: 2L86) [43], and (**D**) long-and-straight helix (PDB: 2KB8) [44]; (**E**) secondary structure of rIAPP: (PDB: 2KJ7) [45]; (**F**) secondary structure of rIAPP complexed with the AMY2 receptor (PDB: 7TYX) [24]. (**B**–**F**) Red: α-helix, Blue: 3_10_-helix, Cyan: helix distortion, and Green: random coil.

The IAPP sequence found in humans has a high propensity to aggregate under physiological pH owing to the presence of two hydrophobic segments (12–17 and 23–27), several Asn residues that can participate in hydrogen-bonded ladders, and only two charged residues (Lys1 and Arg11) (Figure 1A) [18,19,20,21]. The regions 1–18 and 31–37, which are well conserved across evolution [22], are known to be critical for the binding and activation of cognate GPCRs [23,24,25]. In contrast, the central 20–29 region of IAPP is more variable amongst species, which accounts for the fact that IAPP from several species do not self-assemble into amyloids [26,27]. For instance, the sequence found in rodents differs by six residues from human IAPP (Figure 1B) and includes three Pro residues within the 20–29 amyloidogenic core that prevent aggregation into amyloid fibrils [26,27]. Furthermore, rodent IAPP has an Arg at position 18, in contrast to an His in the human sequence. It has been shown that the protonation of the imidazole ring of His18 slows down IAPP oligomerization and aggregation [28,29], likely contributing to the prevention of amyloid fibrils in the acidic pH (≅5.5) of the secretory granules [30]. In contrast, the mutation S20G that is present in the Japanese population and predisposes them to develop early onset T2D is known to accelerate IAPP aggregation [31,32]. Over the last twenty years, numerous thorough mechanistic studies have highlighted the critical contributions of specific residue side chains for IAPP amyloid formation, including Asn14 [33,34], Phe15 [35], Asn21 [20,36,37], Phe23 [35,38], and Ser29 [33,39]. For more details regarding the relationships between primary structure and amyloid aggregation, readers are encouraged to consult the exhaustive recent review by Milardi and colleagues [15].

### 2.1. Secondary Structures

In aqueous solution and under its monomeric state, IAPP mainly exhibits a random coil secondary structure [40], although it populates transient helical segments [41]. Under environments that mimic the cell plasma membrane, such as lipid micelles, detergents, or organic solvents, IAPP readily adopts an α-helical secondary conformation, with its length and position varying according to the environment, including (*i*) short helix [42] (Figure 1B), (*ii*) long-and-kinked helix [43] (Figure 1C), and (*iii*) long-and-straight helix [44] (Figure 1D). The presence of Pro residues within the 20-29 domain of rat IAPP induces a long-and-kinked α-helix in the presence of zwitterionic micelles composed of dodecylphosphocholine (DOPC) [45] (Figure 1E). Interestingly, cryo-EM characterization of the complex between the AMY2 receptor and rIAPP revealed that the peptide adopts a short α-helix spanning from Cys7 to Ser19 upon its insertion in the binding pocket of the receptor [24] (Figure 1F), indicating that the helical secondary structure is critical for the biological activity of the peptide hormone.

### 2.2. Molecular Pharmacology

IAPP mediates its biological activities through its specific binding to the calcitonin receptor (CTR) that can dimerize with receptor activity-modifying proteins (RAMPs), altering the specificity towards the different peptide hormones of the calcitonin family [46]. IAPP is known to bind the CTR alone or associated with three different RAMPs, forming the AMY receptors 1 to 3 [47]. Members of the calcitonin peptide family share three main pharmacophoric structural elements: the amidated C-terminus, the disulfide bridge in the N-terminal region, and the helical conformation spanning across the central region that allows the precise positioning of the residue side chains that are critical for receptor activation [48]. For instance, the C-terminal amide was shown to interact closely with Ser129 and the Trp79 of the CTR receptor [49], allowing the perfect alignment of the peptide within the binding pocket [23]. The helical conformation is critical for receptor activation [24,25], as shown by the cryo-EM of the rIAPP peptide with AMY receptors [24]. Moreover, the contributions of residue side chains of the N-terminal region were investigated through Ala-Scan [50]. This study revealed that when Lys1, Asn3, and Thr4 are substituted with an alanine, IAPP binds the receptor and stays bioactive. In contrast, when Thr6 is replaced by Ala, the resulting IAPP analog is no longer active and binds the receptor with a reduced affinity [49,50]. Furthermore, Leu12 was shown to be critical for the interaction with the first transmembrane α-helix (TM1) of AMY1, as its substitution makes the peptide unable to bind the receptor [49]. The amidated C-terminal region is thought to facilitate the binding of IAPP to the receptor [49,51]. Indeed, when the amidated C-terminus is modified to a carboxylate, the peptide does not bind the AMY1 nor the AMY3 [49]. However, when Tyr37 is substituted by a Phe, there is almost no change in the binding of the peptide to its receptors [49], emphasizing the importance of the amidated C-terminus, more than the hydroxyl group of the C-terminal residue.

## 3. Mechanisms of Amyloid Formation

Pancreatic cell degeneration associated with the tissue deposition of IAPP was initially attributed to the fibrils owing to their presence in the vicinity of the β-pancreatic cells. Cytotoxicity was observed following local inflammation by the increase in the quantity of free radicals [52]. In addition, it was reported that in the presence of a significant amount of insoluble amyloid fibrils within a tissue, the pressure exerted on the organ can lead to cellular death [11]. Nonetheless, most recent studies conducted with IAPP and other amyloidogenic polypeptides have shown that the cytotoxicity is mainly related to oligomers and pre-fibrillar aggregates [53]. Among the several proposed mechanisms, plasma membrane disruption is the most commonly accepted. Three mechanisms of membrane perturbation have been described for IAPP: (*i*) pore formation by helical oligomers, (*ii*) destruction of the lipid bilayer by the formation of oligomers on the outer leaflet, and (*iii*) assembly of oligomers into fibrils on the cell surface [54]. In addition, other cellular mechanisms, linked to membrane disruption or not, have also been proposed, including oxidative stress, apoptosis, and mitochondrial dysfunction. For more detailed information regarding the toxicity of IAPP oligomers, the readers are encouraged to consult these exhaustive reviews [54,55]. Overall, the mechanisms of toxicity of IAPP aggregates remain complex and are highly dependent on the nature of the proteospecies of the amyloid cascade, requiring a deep understanding of the process of amyloid self-assembly at the molecular level.

**Figure 2:**
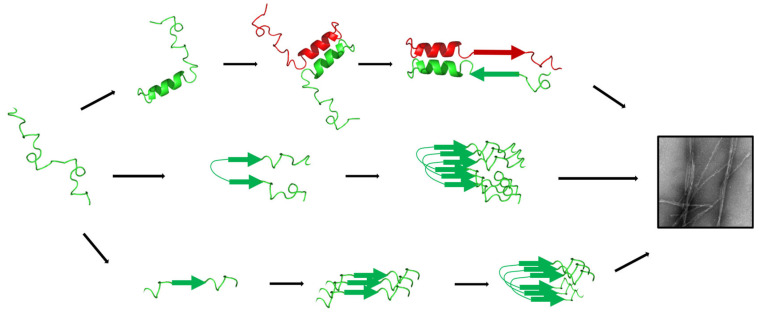
Schematic representation of oligomerization. (α-helix model: top) IAPP adopts a helicoidal conformation, and the 5–20 region of two adjacent monomers forms a coil-coiled conformation, leading to the formation of a β-sheet structure. (β-hairpin model: middle) The segments 9–17 and 23–33 stack together into two β-strands to adopt a U-shape β-hairpin. (stack of β-strand model: bottom) The hydrophobic segment 21–27 of adjacent monomers stack on each other, before a concerted conformational shift into β-hairpin, leading to amyloid elongation.

IAPP amyloid self-assembly is usually ascribed as a nucleation-dependent polymerization, also known as nucleation–elongation polymerization, characterized with three distinctive phases: (*i*) lag phase, (*ii*) elongation phase, and (*iii*) saturation phase [56,57]. The lag phase is characterized by the fast equilibrium between monomers and oligomers that undergo an infinite secondary and quaternary conformational rearrangements [58]. The elongation phase begins with the formation of competent nuclei, leading to the exponential growth of amyloid fibrils by the addition of monomers and/or oligomers to the growing end of the protofibrils, with the occurrence of secondary nucleation that implies fibril fragmentation and fibril-catalyzed nucleation [56,59]. As nuclei are transient and structurally unstable [60], it is difficult to identify what conformation(s) trigger the elongation phase. This information is particularly important to design inhibitors of amyloid formation that can interfere with the early steps of amyloid formation. In fact, numerous studies have revealed that well-defined amyloid fibrils are weakly cytotoxic, while oligomers and pre-fibrillar assemblies that populate the lag and elongation phases are the major culprit proteospecies [37,61,62]. Finally, the saturation phase implicates the reorganization between protofilaments packing into different mesoscopic morphologies of fibrils and is characterized by a low and constant concentration of monomers and oligomeric proteospecies. As proposed for other amyloidogenic polypeptides [63], additional kinetic models of amyloid formation could also describe IAPP self-assembly, including the nucleated conformational conversion [64] and the downhill polymerization [65,66]. Notwithstanding the different kinetic models that best described amyloid formation, IAPP self-assembly remains a highly dynamic process with an infinite diversity of secondary and quaternary structures and a large array of concurrent off- and on-pathways. Thus, it is very challenging to characterize the conformations of the nucleating oligomers and the structures of the prefibrillar proteotoxic species [67]. Over the last two decades, three main hypotheses have been proposed regarding the conformation of the on-pathway oligomers and/or competent nuclei: (*i*) the helical intermediate model, (*ii*) the β-hairpin model, and (*iii*) the stack of β-sheet model (Figure 2).

### 3.1. Role of Helical Intermediates in Nucleus Formation

The first indication that the secondary helical conformation of IAPP could contribute to amyloid formation originates from the observations that environments, or macromolecules, that promote helical folding [68], such as organic solvents [69], lipid membranes [44,70,71,72], and glycosaminoglycans [73], also accelerate amyloid formation. Under these amyloidogenic-prone conditions, IAPP first shifts from a primarily random coil structure to an α-helix, before converting into a β-sheet-rich conformation associated with fibril formation, supporting the hypothesis that helical intermediates could be an on-pathway to fibril formation [70,71,74]. By following the kinetics of amyloid formation using two-dimensional infrared (2D IR) spectroscopy, it was reported that the disappearance of the disordered structures was first accompanied by the emergence of helical conformations, which preceded β-sheet formation [69]. More recently, it was observed by Raman and IR spectroscopy that oligomers have a considerable content of α-helix, in contrast to larger aggregates that are largely composed of β-sheets [75]. Indeed, a α-helix including residues Leu12 and Ala13 is present in approximately 30% of molecules, just prior to their aggregation into larger proteospecies [74]. According to the ‘helical intermediates’ hypothesis, the N-terminal 5-20 helical segment from adjacent monomers drives IAPP initial self-recognition, leading to the formation of a coiled-coil supramolecular motif [41,68]. This association brings the central and C-terminal amyloidogenic segments of adjacent monomers close, promoting the formation of intermolecular cross-β-sheet assemblies.

However, other studies have suggested that the formation of helical oligomers is not a prerequisite for nucleation and could be an off-pathway to amyloid fibril formation. For instance, the restriction of helical folding through the incorporation of two successive D-residues within the helical region did not affect the kinetics of amyloid formation in aqueous solution, and amyloid self-assembly was even accelerated in the presence of lipid vesicles [76]. Similarly, stabilization of the helical conformation through site-specific modifications, including N21Aib substitution, delayed amyloid formation [36]. More recently, it was reported that the stabilization of IAPP helical conformation by side chain-to-side chain macrocyclization, also known as ‘stapling’, inhibits the aggregation and cytotoxicity of IAPP [77], indicating that α-helix stabilization represents an attractive approach to arrest amyloid formation. Additionally, molecular dynamics (MD) simulation has proposed that upon binding to lipid membranes, the α-helical secondary structure of IAPP needs to be first converted into a β-hairpin to initiate self-association into cross-β-sheet fibrils [28,78,79].

### 3.2. The β-Hairpin Oligomers

By integrating replica exchange molecular dynamics (REMD) with results of ion mobility mass spectroscopy analyses of monomers and oligomers, it was proposed that the competent nuclei of IAPP are enriched in β-strand oligomers with the presence of a β-hairpin secondary conformation [40,80]. Interestingly, in contrast to the non-amyloidogenic rat IAPP, human IAPP exhibits a high proportion of β-hairpin secondary conformation (between 24% and 33%), supporting the contribution of this secondary structure for nucleation [40,80]. Similarly, REMD analyses of amyloidogenic and non-amyloidogenic IAPP sequences revealed that the β-hairpin structure is only observed in the aggregation-prone sequences, suggesting that this conformation could be a direct precursor to the competent nuclei leading to amyloid fibrils, and/or that could participate in the formation of toxic oligomers [81]. According to the β-hairpin model, the two β-strands span between residues Thr9-Val17 and Gly24-Gly33, with the loop involving positions His18 to Phe23 [40,81], a structure that closely resembles the ‘U-shape’ conformation of IAPP within mature amyloid fibrils [82]. This conformation would be stabilized by the non-polar contacts between hydrophobic side chains between Leu12 and Leu27 and a hydrogen bond network of the backbone [83,84]. Interestingly, an engineered β-wrapin protein, HI18, was shown to inhibit IAPP aggregation at substoichiometric concentrations by binding and stabilizing this β-hairpin structure, supporting the critical role of this conformation for nucleation [83]. While the β-hairpin model of competent oligomers suits well to the structure of mature IAPP amyloid fibrils, experimental evidence of this model remains sparse, with most observations emerging from MD simulations.

### 3.3. Oligomers Assembled from Stacks of β-Strands

The Ser20-Ser29 segment of IAPP is well-known to contribute to self-recognition and amyloid formation, as numerous studies have shown that isolated fragments of this domain can assemble into amyloid fibrils [26,85], with the fragments Phe23-Leu27 being the shortest to aggregate [86]. Furthermore, IAPP sequences from different species with low amyloidogenicity differ within the amyloidogenic Ser20-Ser29 region [22,87]. For instance, the rodent sequence differs from the one found in humans by the presence of three Pro residues [26,27], whereas the single substitution I26P is sufficient to inhibit IAPP aggregation [88]. While these early works demonstrated that the hydrophobic Ser20-Ser29 region is critical for IAPP self-recognition into ordered cross-β-sheet quaternary structures and that short peptides originating from this segment are prone to assemble into amyloid structures, this region is mainly located in a disordered loop region in most amyloid fibril structures reported so far [82,89,90,91], questioning its contribution in the nucleation step and/or fibril elongation. Accordingly, Buchanan and colleagues incubated full-length IAPP in the presence of stabilized macrocycles that promote the formation of a β-sheet in each region of IAPP [92]. When the β-sheet is stabilized between residues Arg11-Val17, Phe15-Asn21, Asn26-Val32, and Asn31-Tyr37, the formation of fibril is faster, but when the macrocycle stabilizes the Asn21-Leu27 region in the β-sheet, the lag phase is significantly increased. This observation suggests that the conformational transition of the region Asn21-Leu27 from a β-sheet structure to a loop, or disordered conformation, constitutes the thermodynamically limiting step for the initiation of amyloid elongation [92].

In addition, the side chain of residue Asn21 appears to be important for the switch from oligomers to amyloid fibrils. Substitutions of Asn21 by a Phe, Gln, or Leu make the oligomeric-like conformation very stable, giving the peptide strong cytotoxicity and the inability to assemble into cytocompatible amyloid fibrils [36,37]. Notably, the cytotoxicity observed for the N21Q oligomer-like fibrils has been associated with the solvent exposition of the Phe23, which is buried within the fibrillar state of unmodified IAPP, with the amyloid fibrils being non-toxic [37]. Furthermore, the substitution N21P accelerated the formation of non-toxic fibrils, indicating that this position acts as a molecular hinge promoting the conversion from oligomers to cross-β-sheet assemblies [36]. Similarly, by FTIR spectroscopy performed with site-specific isotopically labelled IAPP within the segment Phe23-Leu27, it was observed that the central Phe23-Leu27 region of IAPP drives the initial self-recognition of IAPP towards the formation of fibrillar structures [36,93]. Furthermore, it has been observed that during oligomerization, the C-terminal region of adjacent monomers gets close together, while the N-terminal region only gets close when the fibrils are present, in agreement with the stacks of β-sheet hypothesis [94].

## 4. Molecular Architectures of Amyloid Fibrils

**Figure 3:**
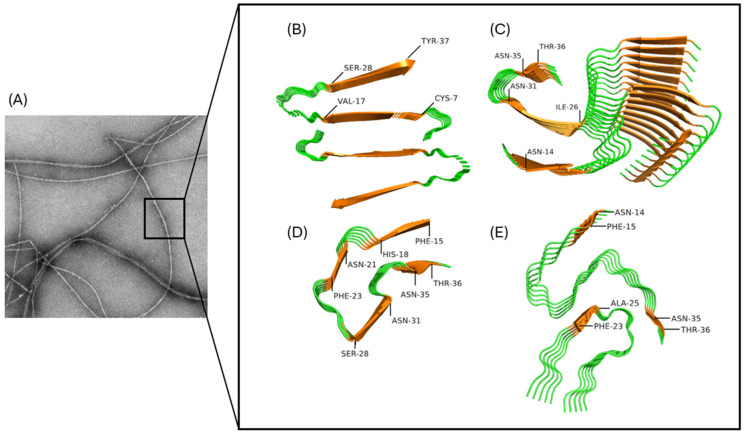
Structure of IAPP amyloid fibrils. (**A**) Transmission electronic microscopy of an amyloid fibril of IAPP, (**B**) U-shaped fibril [82], (**C**) S-shaped fibril (PDB:6Y1A) [89], (**D**) fibrils obtained from the S20G IAPP mutant (PDB: 6ZRQ) [90], and (**E**) polymorphism 1 of IAPP fibrils grown from extracted fibrils of patients afflicted with T2D (PBD: 7M61) [91]. (**B**–**E**) β-strands are indicated in orange, and disordered regions are shown in green.

At the mesoscopic level, IAPP amyloid fibrils appear as long and unbranched filaments of diameters ranging between 5 and 15 nm and lengths that could reach up to 1 µm (Figure 3A). Different atomic-level structures of IAPP amyloid fibrils have been reported, and this polymorphism of supramolecular organization highlights the complexity of the self-assembly process encompassing multiple aggregation pathways [82,89,91,95,96,97,98]. Nonetheless, these structures all share the prototypical cross-β-sheet quaternary conformation that is characterized by the stack of β-strands that are running perpendicularly to the fibril axis. This molecular organization led to an X-ray diffraction pattern characterized by a clear reflection of 4.7Å along the direction of the fibril [99] and the capacity to bind fluorogenic dyes, such as ThT or Congo Red [100]. The first structural models of IAPP fibrils inferred by solid-state nuclear magnetic resonance (ssNMR) and electron paramagnetic resonance (EPR) revealed that each monomer adopts a U-shaped structure and contains two β-strands connected by a loop [82,96] (Figure 3B). More recently, structures obtained by cryo-EM revealed that IAPP monomers adopt a S shape within the fibrils, with each peptide molecule composed of three β-strands [89,90,91] (Figure 3C). Moreover, cryo-EM analysis of amyloids assembled with the S20G mutant of IAPP, which contributes to early-onset T2D [31], revealed fibrils with different morphology characterized by four β-strands per IAPP molecule [90] (Figure 3D). Interestingly, the extraction of amyloid fibrils from patients with T2D and their amplification by seeding with a synthetic peptide also revealed high polymorphism, with four structures arising from distinct packing of the protofilaments [91] (Figure 3E).

## 5. Chemical Strategies to Prevent IAPP Amyloid Formation

Strategies to prevent amyloid tissue deposition and/or the cellular degeneration associated with the formation of transient cytotoxic oligomers can be divided into four categories: (*i*) monomer stabilization, (*ii*) inhibition of oligomerization via prevention of key interactions, (*iii*) inhibition of fibril elongation/formation, and (*iv*) fibril destabilization/destruction [101]. The lack of clear, detailed information regarding the mechanisms of aggregation of amyloidogenic polypeptides and the structures of prefibrillar aggregates makes the development of effective treatments particularly challenging. Nevertheless, some compounds are currently used in the clinics to treat amyloidosis linked to the misfolding of transthyretin (TTR) and the aggregation of the amyloid β (Aβ) peptide. In the case of TTR, tafamidis stabilizes the tetrameric form of the protein, which inhibits tetramer dissociation into aggregation-prone monomers [102]. However, the drug is not effective in disassembling TTR fibrils and requires early detection of the disease, which is not the case for the majority of patients [103]. Furthermore, unlike IAPP, TTR has a well-defined native structure, which allows the rational design of drugs that intercalate into the tetrameric structure of TTR [102]. Recently, the monoclonal antibodies lecanemab, aducanemab, and gantenerumab, which bind to Aβ protofibrils and amyloid plaques, have been approved by the FDA [104]. While these therapeutic antibodies mainly target the aggregated form of Aβ, these antibodies can also bind, to a certain extent, the monomeric precursor. Nonetheless, Aβ is a by-product of the amyloid precursor protein, and, in contrast to IAPP, its physiological roles remain debatable [105]. Thus, considering the important biological activities of IAPP, the usage of monoclonal antibodies that target pancreatic amyloid deposits and/or prefibrillar aggregates should be done with caution in order not to target the monomeric and biologically active form of this peptide hormone.

### 5.1. Small Molecules as Inhibitors of IAPP Amyloid Formation

Among small molecules, polyphenols have shown persistent capacity to inhibit, or interfere, with the amyloid formation of several amyloidogenic polypeptides associated with disease states, including Aβ, TTR, α-synuclein, and Tau [106,107,108]. These natural molecules are abundantly present in plants and are well-known for their positive properties to human health, including anti-inflammatory, anti-cancer, cardio-protective, immunomodulatory, neuroprotective, antibacterial, and/or antiviral [109]. Notably, several studies have reported that epigallocatechin gallate (EGCG), a polyphenol found in green tea, inhibits IAPP amyloid formation and associated toxicity by interacting with the different proteospecies of the amyloid cascade [110]. Moreover, it has been shown that EGCG can disassemble or remodel IAPP amyloid fibrils [110,111]. More recently, it has been observed that the polyphenolic gallotannin corilagin, which has been identified in over 50 plants [112], inhibits IAPP amyloid formation by primarily targeting secondary nucleation and promoting off-pathway aggregation into cytocompatible clusters [113].

Another natural molecule, yakuchinone B, showed inhibitory activity against IAPP aggregation [114]. Analogs of this molecule were designed, and based on ThT fluorescence kinetics and molecular docking, it was proposed that the catechol motif contributes to the formation of hydrogen bonds with IAPP Ser19 and Asn22 side chains [114]. Such interactions observed between IAPP and the catechol could be transposed to other polyphenols due to the presence of numerous hydroxyl groups. While polyphenols have shown interesting properties as inhibitors of IAPP amyloid self-assembly, their promiscuous binding to multiple protein targets, their poor drug-like properties, and the low understanding of their mechanisms of action preclude their usage as anti-amyloid drugs in the context of T2D. Moreover, polyphenolic compounds can interfere with ThT fluorescence, often used to screen for inhibitors of amyloid formation, which could lead to false positives. For instance, while the polyphenol resveratrol found in red wine has been initially identified as a potent inhibitor of IAPP amyloid formation based on ThT fluorescence [115], alternative biophysical characterization revealed that resveratrol remains a weak inhibitor [94]. This observation demonstrates that precautions should be taken when assessing inhibition of amyloid formation solely based on ThT kinetics.

The rational design of small molecules that specifically target and/or stabilize a given IAPP conformation has shown some success (Table 1), albeit this strategy is challenging owing to the large secondary and quaternary conformational ensemble of IAPP during the amyloidogenic cascade. For instance, foldamers, which are molecules that mimic protein conformation [116], have been designed to stabilize the helicoidal conformation of IAPP. Notably, quinoline, pyridyl, and peptoid-like polycarboxylated foldamers have shown the capacity to prevent the aggregation of IAPP in the presence of lipid membranes, known to promote IAPP helical folding [117,118,119]. However, these compounds only showed an inhibitory effect in the presence of lipid vesicles, while an acceleration of IAPP fibrillogenesis was observed in lipid-free conditions. Inspired by this strategy, a small library of 2,5-diaryl substituted thiophenes, designed as helix mimetics, has been evaluated as an inhibitor of IAPP amyloid formation, with some derivatives showing strong inhibition in the absence of lipid membranes [120]. Moreover, a di-phenyl pyrazole compound has shown promising results by stabilizing clusters of random coil IAPP and by disassembling IAPP amyloid fibrils [121]. Recently, another oligopyridylamide scaffold has shown the capacity to inhibit IAPP aggregation at a substoichiometric ratio. The naphtalimide-oligopyridylamide stabilized the N-terminal helicoidal conformation of the monomer and was able to disassemble amyloid fibrils [122]. Finally, metformin, a biguanide developed as an antidiabetic drug owing to its capacity to activate the AMP-activated protein kinase [123], was also shown to reduce the amount of IAPP aggregates found in the pancreas [124]. Recently, metformin was tested as an IAPP amyloid inhibitor, and it was observed that the small molecule reduces the amount of well-defined fibrils by promoting amorphous aggregation [125].

**Table 1:** Small molecules acting as inhibitors of IAPP amyloid formation.

Inhibitors	Proposed Mechanisms of Action and/or Experimental Observations
Epigallocatechin gallate	Inhibition of amyloid formation and remodeling of amyloid fibrils [110]
Corilagin	Inhibition of secondary nucleation and fibril elongation by targeting oligomers and/or pre-fibrillar aggregates [113]
Yakuchinone B	Inhibition of IAPP aggregation by binding to monomeric IAPP [114]
Tetraquinoline	Inhibition of primary nucleation and fibril formation by targeting monomers and/or oligomers [119]
Pentaquinoline	Interaction with helical oligomers in the presence of lipid vesicles [117]
Pyridylamides	Inhibition of fibril formation by targeting monomers and/or low-order oligomers [118]
2,5-diarylated thiophenes	Partial inhibition of primary nucleation and amyloid elongation [120]
Oligopyridylamide	Inhibition of fibril formation by targeting monomers and disassembly of fibrils [122]
Anle145c	Stabilization of non-toxic oligomers and disassembly of fibrils into non-cytotoxic oligomers [121]
Metformin	Reduction of IAPP insoluble aggregates of IAPP in the pancreas and formation of amorphous aggregates [124,125]

### 5.2. Peptides as Inhibitors of IAPP Amyloid Formation

Several synthetic peptides inspired, or not, by the IAPP sequence have been identified as potent inhibitors of amyloid formation (Table 2). To inhibit amyloid self-assembly, one strategy resides in the incorporation of N-methylated amino acids within the amyloidogenic core. The substitution of the hydrogen of the alpha nitrogen atom by a CH_3_ prevents the formation of the hydrogen bond ladder that is essential for monomer stacking and protofilament elongation. Short N-methylated peptides encompassing the IAPP amyloidogenic core were synthetized. N-methylated truncated derivatives of the SNNFGAILSS segment were shown to reduce IAPP cytotoxicity by favoring the formation of cytocompatible aggregates to the detriment of the elongated fibrils [126]. Recently, the N-methylated fragment 15–40 of the Aβ peptide was evaluated to determine crucial interactions with IAPP in order to develop inhibitors of the aggregation [127]. In addition to the N-methylation strategy, the residues in the tripeptide Aβ_(24–26)_, known to form a loop in the final structure of the fibrils, were substituted with larger hydrophobic residues to improve interactions, enabling β-sheet interactions. These tripeptides showed the capacity to co-assemble with IAPP, reducing cytotoxicity and allowing the proteolytic degradation [128].

Macrocyclic peptides inspired by the β-stack oligomer model were designed. These macrocycles were composed of one six-residue recognition strand and one blocking strand that includes a non-natural amino acid, which will preclude the formation of intermolecular hydrogen bonds. Among these macrocycles, only the macrocycle with the Ser20-Leu27 region as the recognition β-strand showed the capacity to delay the primary nucleation, suggesting that the stabilization of the Phe23-Leu27 region as a β-strand increases the free energy barrier to reach the quaternary fold [92]. The non-aggregative properties of the rodent IAPP sequence have also been explored as a lead in designing inhibitors. Based on a Pro-scan of the decapeptide Ser20-Ser29, a full-length IAPP analog with a unique proline substitution at position 26, i.e., I26P, demonstrated potent inhibition of unmodified IAPP amyloid aggregation [88].

As discussed above, some studies have proposed that the helical conformation of IAPP could be an off-pathway to amyloid formation [76,129], and this hypothesis was exploited to rationally conceive peptide inhibitors of IAPP aggregation based on self-recognition. To prevent the formation of β-sheet and promote helical folding, Aib residues were introduced into the IAPP sequences [130]. In fact, it has been previously shown that the presence of an Aib amino acid in peptide sequences, including the Aβ peptide, induces a 3_10_ helicoidal conformation [131,132]. Three peptide fragments (i.e., Ala13-Val17, Ala13-His18, and Ala13-Ser20) were synthesized, and the Ala and Leu residues were substituted with the β-breaker and helical-promoter Aib. FTIR analysis revealed that the Aib-containing peptides adopt a 3_10_ helix. At a molar ratio of 10:1, the Aib-constrained peptides inhibited IAPP amyloid aggregation, with the Aib13-His18 peptide showing the more pronounced effect [130]. Recently, a small library of IAPP peptides helically stapled with an intramolecular triazole between residue side chains at positions *i* and *i* + *4* was prepared and evaluated as inhibitors of amyloid assembly. Among these macrocyclic derivatives, c[Pra^20^-AzK^24^]IAPP showed the more pronounced inhibitory effect on IAPP fibrillization, likely by forming a coiled-coil complex with the unmodified IAPP peptide [77]. Interestingly, it was reported that the side chain-to-side chain stapling strategy, including lactamization, azide-alkyne click chemistry, and thioether link, has a strong impact on helical stabilization and, thus, on the inhibition of amyloid formation [133].

In short pentapeptide derivatives, residues Phe23 and Ile26 were successively substituted with the non-natural residue α,β-dehydrophenylalanine (ΔF), containing a Cα=Cβ motif, which promotes a β-turn to the polypeptide backbone. The pentapeptide [I26ΔF]IAPP_(23–27)_ showed significant potency to inhibit the aggregation of IAPP, probably by interacting with the amyloidogenic core. In agreement with the hypothesis of helical oligomer intermediates, it was suggested that these compounds prevent the coiled-coil formation observed in the early stage of the aggregation process [134]. In addition, a small library of β-hairpin derivatives was developed based on a piperidine-pyrrolidine β-turn inducer linked to different types of molecular arms: pentapeptide, tripeptide, and/or α/aza/aza/pseudotripeptide. Longer peptides provided enhanced inhibition of IAPP amyloid aggregation [135].

During secretion, IAPP is trafficked into the secretory vesicles of pancreatic β-cells with insulin at a molar ratio of 1:100 (IAPP:insulin), suggesting that insulin could prevent IAPP aggregation. As a matter of fact, it was observed that insulin inhibits IAPP amyloidogenesis, even at substoichiometric ratios, and this effect was mainly associated with a sharp delay in primary nucleation [136]. By molecular simulation and experimental validation by chemical crosslinking, it was observed that the helical domain of the chain B of insulin (Ser9-Phe24) interacts with the central region of IAPP (Ala8-Phe23), stabilizing IAPP in a non-aggregation helical conformation. Notably, this interaction between the two glucomodulatory peptides was stabilized by an electrostatic bridge between Arg11 of IAPP and Glu13 of insulin, as well as hydrophobic interactions [137]. Furthermore, decapeptide fragments from the insulin B chain were evaluated as inhibitors of IAPP aggregation, with fragment 9–20 being the more potent amyloid inhibitor [138].

Recently, a small library of glycosylated derivatives of the 12–27 amyloidogenic region of IAPP was designed. In line with observations that glycosylated Aβ fragments are markedly reduced in patients with AD [139], the glycosylated peptide highlights the functional relevance of this modification in amyloid regulation. The engineered oligopeptides, comprising two IAPP hot segments linked by a glycosylated tripeptide spacer, markedly delayed or even completely suppressed IAPP aggregation. Notably, library screening identified S^N^S as the most potent inhibitor. While all glycosylated constructs shared a common mechanism by interfering with β-sheet nucleation of IAPP monomers, S^N^S achieved the more pronounced inhibitory efficacy. It can be explained by the steric hindrance created by the sugar-blocking interactions between two monomers [140].

**Table 2:** Peptides as inhibitors for IAPP aggregation.

Inhibitors	Proposed Mechanisms of Action and/or Experimental Observations
[(N-Me)G24, (N-Me)I26]IAPP_(20–29)_	Inhibition of the formation of β-sheet structure [126]
[(N-Me)V18, (N-Me)Phe20, V24Nle, G25Nle, S26Nle, M35Nle]Aβ_(15–40)_	Delay of primary nucleation by binding to nucleus/oligomers and formation of fibrils that can be eliminated by proteolytic cleavage [128]
cyclo[δOrn^8^-δOrn^15^]NNFGAILKF * HaoYV	Stabilization of the monomer in a non-aggregating conformation [92]
[I26P]IAPP	Inhibition of IAPP amyloid formation by interfering with fibril elongation [88]
[A13Aib, L16Aib]IAPP_(13–18)_	Inhibition of IAPP amyloid formation [130]
c[Pra^20^; AzK^24^]IAPP	Delay in IAPP amyloid formation by forming coiled-coil assemblies mainly by binding to monomers [77]
[I26ΔF]IAPP(23–27)	Stabilization of the IAPP monomer into an helicoidal conformation likely by interacting with monomers [134]
H_2_NAIL[piperidine-pyrolidine-β-turn]FaLaVCOCH_3_	Delay in primary nucleation and inhibition of amyloid fibril elongation [135]
Insulin	Binding and stabilization of the monomer in an non-aggregating helicoidal conformation [137]
[^N^S20S]IAPP(12–27)	Blockage of the interactions between two monomers by binding interactions [140]

(*) Hao = (5-HO_2_CCONH-2-MeO-C_6_H_3_-CONHNH_2_), unnatural amino acid composed of a hydrazine, 5-amino-2-methoxybenzoic acid, and oxalic acid groups [141]. ^N^S = N-acetylglycosamine-Serine [140].

### 5.3. Passive Immunotherapy with Antibodies

Over the last decade, the generation of antibodies (Abs) that specifically recognize proteospecies of the amyloid cascade has been exploited to prevent the aggregation of a given protein and/or to mitigate the toxicity associated with the formation of culprit assemblies. This strategy led to the FDA approval of the monoclonal antibodies (mAbs), aducanumab, lecanemab, and donanemab, which delay the progression of AD by interacting with the aggregated form of the Aβ peptides [142]. These Abs bind their antigens, interfering with secondary nucleation and preventing fibril elongation. Moreover, the amyloid-targeting Abs enable the degradation of the targeted proteospecies through microglial cell-mediated phagocytosis [142]. The first Abs specifically generated against IAPP aggregation were obtained using mouse sera collected after intraperitoneal injection of IAPP oligomers, which were prepared by a controlled aggregation in the presence of the detergent SDS (Table 3). Moreover, anti-IAPP Abs were generated following subcutaneous immunization with a preparation of IAPP attached to a multi-subunit coat protein from the bacteriophage Qβ, a virus-like particle often employed as a nanoscaffold in vaccines [143]. For both studies, inoculation of the immunized mice sera to hIAPP transgenic mice led to a decrease in blood sugar concentration and a reduction in IAPP aggregates observed in the pancreas [143,144]. More recently, mAbs were generated by preparing a hybridoma from immunized mice with two different antigen inoculations: (*i*) IAPP attached to a VLP [145] and (*ii*) IAPP protofibrils stabilized with the NUCB1 chaperone [146]. In addition, serum from a healthy elderly individual was also used to identify Abs that recognize specifically IAPP aggregates [147]. Upon hybridoma preparation and purification of the mAbs, these antibodies were shown to inhibit primary nucleation and/or fibril elongation [145,146,147]. After the injection of these mAbs in diabetic transgenic mice, a decrease in fibril deposition in the pancreatic islets was also observed [145,146,147].

**Table 3:** Antibodies inhibiting IAPP aggregation and amyloid deposition.

Antibodies	Mechanisms of Action
Oligomer-specific antibody	Reduction of IAPP aggregation in the pancreas and increased production of insulin [144]
m81	Recognition of oligomers and amyloid fibrils and inhibition of IAPP oligomerization [143,145]
07G10 and 10H04	Recognition of IAPP protofibrils and inhibition of amyloid formation [146]
α-IAPP-O	Recognition of transient prefibrillar oligomers and inhibition of primary nucleation [147]

### 5.4. Proteins as Inhibitors of IAPP Amyloid Formation

Over the last two decades, several proteins, including chaperones, have been shown to inhibit amyloid aggregation. These proteins often have an inhibitory effect on amyloid self-assembly at substoichiometric molar ratios, with several of them having a specific domain that allows interaction and high-affinity binding with aggregation-prone regions. This constitutes a potential source of inspiration for the rational design of anti-amyloid drugs targeting IAPP. Particularly, chaperones have demonstrated a noteworthy ability to inhibit IAPP aggregation (Table 4). Studies have shown that in T2D, there is an increased expression of chaperones and, in particular, of heat shock proteins (HSPs) [148]. Among HSPs, Hsp70, Hsp40, and BiP have been shown to increase the lag time of IAPP nucleated polymerization, even when these proteins are used at substoichiometric ratios [149]. Also, the co-chaperone prefoldin, an HSP60, has shown an inhibitory effect on IAPP assembly at substoichiometric levels [150]. The structure of prefoldin is characterized by a β-barrel and tentacles with coiled-coil α-helices, allowing the formation of a cavity in which IAPP binds. ThT kinetics revealed an effect on both primary and secondary nucleation, suggesting that prefoldin is capable of recognizing monomers as well as protofibrils [150].

Interestingly, the BRICHOS domain, which is found in a number of proteins, has shown persistent ability to bind to amyloid fibrils and to modulate the process of amyloid formation of several amyloidogenic proteins, such as α-synuclein, Aβ peptide, and Tau [151,152,153]. For instance, the protein Bri2 delayed the nucleation of IAPP, and this inhibitory activity was dependent on the presence of its BRICHOS domain [154]. Moreover, by computational simulation, it was revealed that the BRICHOS domain binds preferentially to amyloid fibrils compared to the monomeric peptide through a combination of hydrophobic and electrostatic interactions, as well as hydrogen bonds [155].

Several variants of the apolipoprotein E (ApoE), a lipoprotein associated with the development of Alzheimer’s disease [156], have been evaluated as potential inhibitors of IAPP aggregation. Notably, the ApoE, along with IAPP, is also a component of the amyloid plaques found in the brain of patients afflicted by Alzheimer’s disease [157]. It was reported that ApoE modulates IAPP aggregation at substoichiometric ratios by binding preferentially to the monomer and by altering fibril morphology. ApoE also protected pericyte cells from IAPP-induced cytotoxicity [157]. TTR, a plasma-circulating protein whose aggregation is associated with systemic amyloidosis, has been previously reported as a potent inhibitor of Aβ peptide self-assembly and cytotoxicity [158]. More recently, it was observed that TTR, under its monomeric and tetrameric isoforms, stabilizes transient oligomeric proteospecies, thereby prolonging the lag phase by precluding IAPP nucleation and/or elongation phase, even when employed at very low substoichiometric concentrations [159]. Finally, the domain B of the A protein of Staphylococcus aureus, which adopts the affibody structure, was used to engineer a β-wrap protein characterized by a bundle of three α-helices that is known to specifically recognize the β-hairpin motif. The resulting β-wrap affibody protein, HI18, presents a cavity composed of β-helices and β-sheets that enable specific recognition. It was observed that the HI18 protein inhibits IAPP amyloid aggregation [160] by stabilizing the monomer into a β-hairpin conformation [83].

**Table 4:** Proteins as inhibitors and modulators of IAPP amyloid formation.

Inhibitors	Proposed Mechanisms and Experimental Observations
Hsp 40, Hsp70, Grp78	Increase of the lag phase and decrease of amyloid loads by targeting oligomers [149]
HI18	Binding to monomers and oligomers [83]
Bri2 BRICHOS	Inhibition of IAPP aggregation at low molecular ratio by preferentially interacting with fibrils [154,155]
ApoE	Interference with primary nucleation and amyloid fibril elongation [157]
TTR	Elongation of the lag phase by binding to monomer and prefibrillar aggregates [159]
Prefoldin	Inhibition of primary and secondary nucleation [150]

### 5.5. Alternative Chemical Identities Inhibiting IAPP Amyloid Formation

A diversity of nanostructures, such as inorganic nanoparticles, dendrimers, and DNA origami, have also shown efficacy to inhibit IAPP amyloid formation and associated cytotoxicity (Table 5). For instance, 40 nm copolymeric nanoparticles composed of a mixture of N-isopropylacrylamide:N-tert-butyl-acrylamide (NiPAM:BAM) at different ratios were evaluated as modulators of IAPP amyloidogenesis [161]. Among the different NiPAM:BAM nanoparticles, only those composed of molar ratios of 85:15 and 100:0 (NiPAM:BAM) showed inhibitory activity on IAPP amyloid formation [161]. These effects were ascribed to hydrogen bond interactions between the peptide and the nanoparticles that preclude the formation of the hydrogen-bonded ladder of amyloid protofilament. This interaction is promoted by the diminution of the solvation free energy due to the presence of the tert-butyl group found in NiPAM [161].

Recently, gold nanoparticles grafted with aminobenzoic acid (Au@ABA), mercaptobenzoic acid (Au@MBA), and ethynylbenzoic acid (Au@EBA) groups have been used as potential amyloid inhibitors. Only the Au@ABA nanoparticles showed an inhibition of IAPP aggregation, mainly by stabilizing IAPP into a helical conformation and by promoting the formation of peptide aggregates adsorbed on the nanoparticles [162]. Other carbon-based nanoparticles have also shown interesting properties of inhibiting protein aggregation. REMD has shown that hydroxylated single-walled carbon nanotubes (SWCNT-OHs) could stabilize IAPP into a coiled-coil conformation, decreasing the content of β-sheets [163]. Based on these computational observations, SWCNT-OHs were evaluated as inhibitors of IAPP aggregation, and a decrease in ThT fluorescence was observed, which was accompanied by an alteration of the secondary structure observed in circular dichroism spectroscopy [163]. TEM images showed that the IAPP fibrils assembled in the presence of SWCNT-OH nanotubes were shorter and less abundant, compared to those assembled in the absence of carbon nanotubes [163]. Ironoxide nanoparticles (IONPs) decorated with β-casein, a protein found in milk, also demonstrated strong inhibition of IAPP amyloid formation by promoting the formation of amorphous aggregates, as observed by TEM [164].

Dendrimers, which are branched polymeric molecules, were also examined as potential inhibitors of IAPP aggregation. While neutral and cationic dendrimers showed no impact on the aggregation process, dendrimers substituted with carboxylate groups accelerated IAPP aggregation. Interestingly, low-generation sulfated dendrimers showed partial inhibition of aggregation, in contrast to higher-generation sulfated dendrimers [165]. The capacity of the negatively charged polymers to accelerate the aggregation process was also observed by poly(2-hydroxyethyl acrylate) (PHEA) star polymers. The rigid PHEA arms act as a rodlike scaffold, increasing the local concentration of IAPP and accelerating quaternary structural transition [166]. In both cases, anionic dendrimers reduced the cytotoxicity of IAPP [165,166]. More recently, it was reported that 1D, 2D, and 3D double-stranded DNA nanostructures inhibited IAPP amyloid formation and were proficient in disassembling IAPP amyloid fibrils into cytocompatible spherical complexes [167].

**Table 5:** Overview of chemical identities as modulators of IAPP amyloid formation.

Inhibitors	Proposed Mechanisms and Experimental Observations
NiPAM:BAM nanoparticles	Increase of the lag phase and reduction of the elongation phase by interacting with the oligomers [161]
Au@ABA	Partial inhibition of the aggregation by binding preferentially to monomers [162]
SWCNT-OH	Inhibition of the formation of β-sheets and partial inhibition of fibril elongation [163]
βcas IONPS	Inhibition of amyloid aggregation by targeting monomers and oligomers [168]
Anionic dendrimers	Acceleration of aggregation into cytocompatible aggregates [165]
PHEA dendrimer	Acceleration of IAPP aggregation and reduction of toxicity in vivo and ex vivo [166]
DNA nanostructures	Inhibition of the aggregation process and disassembly of amyloid fibrils into cytocompatible spherical complexes [167]

## 6. Design of Non-Aggregating IAPP Peptide Derivatives to Treat Metabolic Disorders

By activating different GPCRs expressed in several tissues, IAPP regulates key physiological functions [169], including the regulation of food intake and glucose homeostasis, highlighting the potential usage of IAPP derivatives as therapeutic agents to treat metabolic disorders, such as T2D and obesity. However, as a peptide therapeutic, IAPP presents some limitations associated with its short half-life, low metabolic stability, and high renal clearance. Above all, its high propensity to aggregate in solution and upon injection precludes its safe usage for drugs. In fact, peptide aggregation generates several problematics with formulation and storage, strongly impacting shelf-stability, therapeutic activity, and the biosafety of peptide therapeutics [170]. As discussed above, N-methylation of IAPP within the 20–29 amyloidogenic core has been exploited to design amyloid inhibitors, and this strategy was also used to develop non-aggregating and bioactive IAPP derivatives (Table 6). Double-N-methylated peptides in the amyloidogenic region were synthesized and showed strong inhibition of IAPP amyloid self-assembly, as well as potent ability to activate IAPP receptors expressed on human breast carcinoma cells MCF-7 [171,172].

The primary sequence of rodent IAPP that contains three Pro residues within the amyloidogenic core has inspired the design of a non-aggregating IAPP drug that is currently used to help patients afflicted with T2D to control their glycemia [173]. Approved by the FDA in 2005, Pramlintide is the first-in-class and the only IAPPomimetic still on the market [174]. Pramlintide has a half-life of 20 to 45 min, which means that it needs to be taken three times a day combined with insulin [175]. Moreover, pramlintide cannot be co-formulated with insulin therapy. Indeed, pramlintide tends to precipitate at a pH higher than 5.5 and needs to be formulated at pH 4 in its acetate form [176,177], which is not suitable for insulin. Interestingly, during the clinical trials, patients who received pramlintide showed significant weight loss, highlighting the potential of IAPP-derived compounds for the treatment of obesity.

As mentioned previously, all three receptor activity-modifying proteins (RAMP1, RAMP2, and RAMP3) can associate with the calcitonin receptor (CTR), resulting in the formation of three distinct amylin receptor subtypes: AMY1, AMY2, and AMY3 [178]. The CTR, when expressed independently at the cell surface, exhibits a distinct pharmacological profile characterized by strong responsiveness to human and non-human calcitonin (CT) peptides, but only weak affinity to IAPP. In contrast, the association of CTR with a RAMP yields an amylin receptor (AMYR) complex that displays high affinity towards IAPP, while showing low responsiveness to calcitonin (hCT). Interestingly, salmon calcitonin (sCT) functions as a non-selective agonist, demonstrating high affinity and efficacy for both CTR and AMYR subtypes. This property makes CT peptides valuable pharmacological tools for probing receptor activation and ligand selectivity within the calcitonin/amylin systems.

Chimeric compounds able to activate the AMY and CT receptors, known as dual amylin and calcitonin receptor agonists (DACRA), were developed in the past years. Although no compound has been approved, numerous long-acting DACRA peptides have been developed, and their sequences are presented in Table 6. With the goal of increasing the half-life of the peptide, the pramlintide sequence has been modified at three residues and lipidated on the N-terminal lysine side-chain to form Cagrilintide (also known as AM833), a compound that was evaluated in clinical trials for treating obesity [179]. Similarly, Davalintide, a peptide combining a portion of rodent IAPP and sCT, both sequences being non-amyloidogenic, with the dual action on both AMYs and CT receptors, has been recently developed [175]. Even if Davalintide showed improved pharmacokinetics, phase 2 clinical trials were discontinued in favor of a mixture of pramlintide and another drug. To further improve peptide bioavailability by limiting elimination by the kidneys, it is possible to conjugate to a fatty acid chain in order to promote the binding of the peptide to serum albumin [180]. The resulting compound, NN1213, a peptide acylated on the epsilon chain of Leu at position 1 using the C20diacid-gGlu-gGlu group (LFa), showed improved pharmacokinetics, supporting the usage of this IAPP derivative as a potential treatment for obesity [180]. Other long-lasting IAPP derivatives reached the clinical phase, such as KBP-042 that reached clinical phase 2 [181]. Most of these IAPP derivatives include a fatty acid to prolong their half-life, such as Petrelintide and Eloralintide. Petrelintide, which reached clinical phase 2, has a lactam bridge between residues Asp2 and Lys7, as well as a N-methylated residue to prevent its amyloid aggregation [182]. Eloralintide, a IAPP derivative with a methylene thioacetate bond and the Lys36 acetylated with two γ-Glu residues, has recently completed phase 2 clinical trials [183].

**Table 6:** Overview of IAPP therapeutic derivatives. Residues of the IAPP sequence are represented in green, while residues from salmon calcitonin are represented in blue. Residues found in the rIAPP sequence are indicated in red.

Peptide	Sequence	Stage of Clinical Development
IAPP	KCNTATCATQRLANFLVHSSNNFGAILSSTNVGSNTY-NH_2_	N.A.
Pramlintide	KCNTATCATQRLANFLVHSSNNFGPILPPTNVGSNTY-NH_2_	FDA approved
sCalcitonin	CSNLSTCVLGKLSQELHKLQTYPRTNTGSGTP-NH_2_	N.A.
Davalintide	KCNTATCVLGRLSQELHRLQTYPRTNTGSNTY-NH_2_	Phase 2
KBP-042	Ac-CSNLSTCVLGKLSQELHKLQTYPRTDVGANAP-NH_2_	Phase 2 [184]
NN1213	(LFa)CNTATCATQRLARHSSPNFGAIPSSTNVGSRTY-NH_2_	Pre-clinical [180]
Petrelintide	[19CD]-isoGlu-RDGTATKATERLA-Aad-FLQRSSFGly(Me)-A-Ile(Me)-LSSTEVGSNT-Hyp-NH_2_	Phase 2 [185]
Eloralintide	(γGlu)-ANTATCATGOrnLAE ((α-Me-Phe))LVRSSN ((N-Me-Asn))FGP(LFa)LPPTGVESNTY-NH_2_	Phase 2 [186]
Cagrilintide	(LFa)KCNTATCATQRLAEFLRHSSNNFGPILPPTNVGSNTP-NH_2_	Phase 3a [187]

(LFa) = Lys C20diacid-gGlu-gGlu; [19CD]-isoGlu = 19-carboxynonadecanoyl covalently attached to the αamino group of an iso-glutamic; Aad = (2S)-2-aminohexanedioic acid; Hyp = 4-Hydroxyproline; N.A. = not applicable.

## 7. Conclusions

Since its initial identification from amyloid deposits of the pancreatic islets in 1987, IAPP has been extensively studied in the context of the etiology of T2D. While the correlation between IAPP deposition as insoluble aggregates in the islets of Langerhans and the progression of β-cell degeneration has been well established, no therapeutic strategies to inhibit this pathological process have yet made it into the clinics. This absence of effective and safe drugs to tackle amyloid deposition likely reflects the complexity of the mechanisms of self-assembly that encompass a large conformational ensemble, precluding the rational design of anti-aggregation agents. While several structures of the soluble monomeric peptide, as well as those of mature amyloid fibrils (thanks to recent advancements in cryo-EM), have been reported over the last two decades, the conformations of the cytotoxic oligomers and other prefibrillar proteospecies remain largely speculative. A more detailed structural characterization of the oligomers, as well as a better understanding of the conformational conversion associated with cell death and pancreatic deposition, is a critical advancement required to develop potent inhibitors. In addition, while it is well established that IAPP interacts with anionic lipid membranes, in vitro models only partially reflect the complexity of the cell plasma membrane [54]. Moreover, the observation that foldamers inhibit IAPP aggregation exclusively in the presence of membranes is consistent with previous findings obtained in aggregation assays performed with and without glycosaminoglycans. Indeed, the addition of GAGs to the medium was shown to reduce the inhibitory effect of insulin [188]. These observations highlight the limited physiological relevance of simplified in vitro experimental models, which fail to capture the complexity of the environment found in pancreatic β-cells [189], a critical aspect to consider for the development of effective amyloid inhibitors.

Moreover, the key physiological roles of the peptide hormone IAPP, including the control of satiety and food intake, highlight its high potential to treat metabolic disorders. Notably, with the booming of GLP-1-based therapeutics to fight obesity, there is a huge interest in developing IAPP derivatives as therapeutics for diabetes and obesity. This will not only require the identification of potent agonists of the AMY/CT receptors that do not aggregate, but also those that do not co-assemble with the endogenous IAPP into pancreatic amyloid fibrils. Again, understanding how IAPP secondary and quaternary conformational ensembles are modulated by interactions with the biological environment remains critical to develop potent, specific, stable, and non-aggregating IAPP derivatives.

## Data Availability

No new data reported.
